# Demographic Characteristics and Clinical Outcome of Work-related Open Globe Injuries in the Most Industrialised Region of Turkey

**DOI:** 10.4274/tjo.81598

**Published:** 2017-01-17

**Authors:** Sertaç Argun Kıvanç, Berna Akova Budak, Emina Skrijelj, Mediha Tok Çevik

**Affiliations:** 1 Uludağ University Faculty of Medicine, Department of Ophthalmology, Bursa, Turkey; 2 Düziçi Sate Hospital, Ophthalmology Clinic, Osmaniye, Turkey

**Keywords:** Blindness, work-related eye injury, open globe injury, visual impairment, work accident

## Abstract

**Objectives:**

To evaluate demographic characteristics and clinical outcomes of work-related open globe injuries in the most industrialized region of Turkey.

**Materials and Methods:**

The demographic and medical records of patients with work-related open globe injuries who presented to the ophthalmology or emergency departments with an official occupational accident report were retrospectively reviewed. Visual acuity categories were defined according to the World Health Organization. The injury types and zones of the open globes were classified according to Birmingham Eye Trauma Terminology System.

**Results:**

Among 479 patients with work-related eye injuries in 5 years, there were 102 eyes of 101 patients with open globe injuries (21%). The mean age of the patients was 34.5±8.9 years with a mean follow-up of 12.5±12.6 months. The injuries peaked in June in the hour between 12:00 and 13:00. Eighty-six percent presented to emergency services within 12 hours after the injury. Twenty-two percent of the patients had been wearing protective eyewear at the time of injury. The open globe injuries were penetrating in 51%, intraocular foreign body in 40%, rupture in 7% and perforation in 2% of the eyes. The most frequent finding was traumatic cataract. Final visual acuity of 33.3% of patients was below 3/60. Seventy-eight percent of patients that had visual acuity worse than 6/18 at presentation had visual acuity of 6/18 or better at final visit. Sixty-three percent of eyes which had injuries involving all 3 zones resulted in phthisis bulbi, enucleation or evisceration.

**Conclusion:**

Work-related open globe injuries may have severe consequences such as visual impairment and blindness among the young male working population in industrialized areas. Nearly half of the occupational open globe injuries resulted in visual impairment and blindness.

## INTRODUCTION

Work-related ocular trauma is an important cause of visual impairment and blindness globally, with a significant socioeconomic impact.^1,2,3,4^ In Turkey, it has been reported that among the work-related injuries admitted to tertiary emergency centers, 3.9-5% were ocular trauma.^5,6^ Open globe injuries were found to be most serious type of ocular trauma with regard to visual outcome.^7,8^ Work-related open globe injuries constitute a considerable proportion of all open globe injuries (28.4-40.3%) in different reports from Turkey.^9,10,11,12^ To our knowledge, the outcome of work-related open globe injuries in Turkey has not been reported before. In this study, our aim was to evaluate demographic characteristics and clinical outcome of work-related open globe injuries in the most industrialized region of Turkey.

## MATERIALS AND METHODS

The medical records of patients with ocular injuries who presented to the Ophthalmology or Emergency departments of Uludağ University between January 2010 and December 2015 with official occupational accident reports were retrospectively reviewed. The patients with work-related open globe injuries were included. Age, sex, information about the injury season and time, the injured eye, the use of protective eyewear, the objects that caused the trauma, initial and follow-up examinations, zone of injury, primary and secondary surgical procedures and outcomes were recorded. Patients were contacted by phone to complete missing information regarding their workplace and use of protective eyewear. We obtained complete information for 85 of 101 patients; information about 16 patients was not available. The initial and final visual acuities (VA) of the patients were divided into visual impairment categories as defined by the World Health Organization (WHO). WHO criteria are accepted worldwide, so we used these criteria for standardization. Blindness is defined as a presenting VA worse than 3/60 or a corresponding visual field loss of less than 10° in the better eye. Visual impairment is defined as a presenting VA of worse than 6/18 and equal to or better than 3/60.^13^ The injury types and zones of the open globes were classified according to the Birmingham Eye Trauma Terminology System.^14^

The SPSS 22 statistical programme (IBM Corp., USA) was used for statistical analysis. Pearson correlation test was used to assess the correlation between initial VA, number of surgeries and final VA. Pearson chi-squared test was used to compare initial and final visual impairment/blindness status. Fisher’s exact test was used to assess the relation between the use of protective eyewear and the presence of protective eyewear in the workplace. Paired samples t-test was used to compare initial and final VA.

## RESULTS

**Figure 1:**
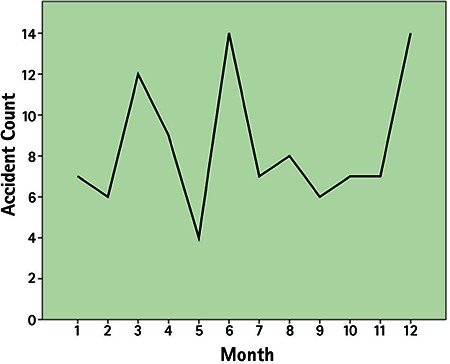
Distribution of work-related open globe injuries by month of occurrence

**Figure 2:**
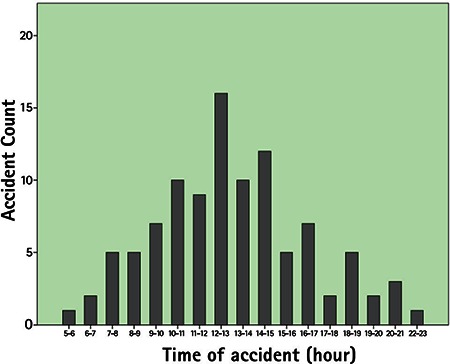
Distribution of work-related open globe injuries by time of occurrence

Among 479 patients with work-related eye injuries seen at Uludağ University in 5 years, there were 102 eyes of 101 patients with open globe injuries (21%). The mean age of the patients was 34.5±8.9 years with a mean follow-up of 12.5±12.6 months. The majority of the injuries occurred in men (99%). About 40% of the injuries took place in June (13.7%), December (12.7%) and March (11.8%) (Figure 1). Right eyes were injured in 39 patients (38.3%), and left eyes were injured in 61 patients (60.4%). Bilateral open globe injury was observed in one patient. Distribution of time of injury over 24 hours (expressed as one hour intervals) is shown in Figure 2. The mean time elapsed from injury to presentation to emergency services was 7.4±13.3 hours. Eighty-six percent presented to emergency services within 12 hours of the injury, 6% presented 12-24 hours after the injury, and 6% presented 24-48 hours after the injury. One patient presented 4 days after the injury and one presented 12 days after the injury. Both had endophthalmitis at initial examination. The objects that caused the injury and the occupations of the patients are given in Table 1 and Table 2.

Nineteen patients (22%) had been wearing protective eyewear at the time of injury, 91.7% (17 patients) of them stated that their workplace provided protective eyewear. Sixty-six patients (78%) had not been wearing protective eyewear at the time of injury, and 39.5% of them (26 patients) stated that their workplace had protective eyewear. The ratio of patients who wore protective eyewear was significantly higher in workplaces that provided protective eyewear (p=0.002), and a significantly higher proportion of large-scale companies provided protective eyewear when compared with small-scale companies (p<0.001). Most of the patients who had been wearing protective eyewear at the time of injury were working in large companies (p=0.019).

Of 101 patients, 9 (9%) had another work-related injury, before or after the work-related open globe injury; these were hand injury in 5 patients, fall in 1 patient, and ocular trauma and corneal foreign body in 3 patients.

The open globe injuries were classified according to Birmingham Eye Trauma Terminology System14 as penetrating in 52 (51%), intraocular foreign body in 41 (40%) and rupture in 7 (7%) and perforation in 2 (2%) eyes. There were 52 zone I (50.9%), 10 zone II (9.9%) and 2 zone III (1.9%) injuries. Fifteen eyes (14.7%) had injuries involving zones I and II, 16 eyes (15.7%) had injuries involving all 3 zones and 7 eyes (6.9%) had injury involving zones II and III. Traumatic cataract and/or crystalline lens dislocation were observed in 73 eyes (71.5%), and other findings were iris injury in 63 eyes (61.8%), hyphema in 47 eyes (46.1%), vitreous hemorrhage and/or posterior segment injury in 40 eyes (39.2%). In 16 (15.7%) cases, there were additional injuries: limb injury in 2, eyelid laceration in 10, multiple lacerations involving the eyebrow and face in 3, and orbital wall and zygomatic fracture in 1 patient. Presenting VA acuity was light perception in 26 (25.7%) and no light perception in 10 (9.9%) patients. The mean logMAR VA of the others was 1.7±1.3. The mean final logMAR VA was 0.6±0.8. Final VA was no light perception in 16 patients (15.8%) and light perception in 7 patients (6.9%). The final VAs were significantly improved compared to initial VA (p<0.001) and there was a significant positive correlation between initial and final VA (r=0,385, p=0,002). The initial and final VAs of the patients and categories of visual impairment according to WHO criteria13 are shown in Table 3. Forty-six percent of patients who were blind at presentation were blind at final visit. Seventy-eight percent of patients that had vision worse than 6/18 at presentation had VA of 6/18 or better at final visit. Three patients who had 6/18 or better VA initially became visually impaired or blind during follow-up (p<0.001). Out of 102 eyes, 83 underwent primary repair. Lens aspiration was performed in 4 eyes and lens aspiration with removal of intralenticular foreign body was performed simultaneously with primary repair in one patient. Nine patients underwent primary repair and removal of foreign bodies in the anterior chamber. Out of 19 eyes that did not require primary repair, 3 eyes without IOFB were treated with bandage contact lens for corneal laceration, 13 eyes underwent pars plana vitrectomy and IOFB removal, 2 underwent phacoemulsification for removal of intralenticular foreign body, and 1 underwent anterior chamber washout for removal of foreign body in the anterior chamber. Primary repair was performed within 12 hours of admission in 60 patients and within 24 hours in 16 patients. Six patients were referred to our department after primary repair elsewhere. The primary surgery was performed after 48 hours in 2 patients. The others who did not require primary repair underwent surgery for IOFB removal after an average of 4.6±3.5 days. Of 43 eyes with IOFB, 29 were in the vitreous or lodged in the retina, 10 were in the anterior chamber, and 2 were intralenticular. Two foreign bodies were actually outside the globe, 1 in the orbit and the other in the ethmoid sinus. Forty of the IOFBs were metallic and 3 were stone. The mean number of surgeries was 1.8±1.0. There was a positive correlation between the number of surgeries performed and logMAR VA (r=0.252, p=0.025). Primary and additional surgeries performed after primary repair are shown in Table 4.

At final visit, 46 eyes were pseudophakic, 26 were phakic, 10 had traumatic cataract, 15 eyes were aphakic, and 4 eyes had undergone evisceration and 1 eye had undergone enucleation. Twelve of the 34 eyes that had VA under 3/60 resulted in phthisis bulbi, enucleation or evisceration. Ten of these 12 eyes had injuries encompassing all zones, and 2 eyes had injuries in zones II and III. The final clinical outcomes of patients with respect to visual impairment and blindness are shown in Table 5.

## DISCUSSION

Both in Turkey and worldwide, eye injuries, especially open globe injuries (either work-related or not), predominantly affect men.^15,16,17,18,19,20^ Accordingly, 99% of the patients in the present study were men. This may be related to the working sectors of the patients. The metalwork and construction sectors, which employ mostly men, were predominant in this study. In this study, the mean age of the subjects was 34.5±8.9 years. A study involving work-related eye injuries from western Turkey reported that the mean age was 28.1±6.5 years, but the majority of the injuries occured in individuals 25-34 years old.^21^ In another study from China, the mean age was reported to be 39.2±11.16 years.^1^ A similar result was reported from northern Thailand.^4^ However, these studies evaluated all occupational eye trauma involving both closed and open globe injuries. Kanoff et al.^22^ reported a mean age of 35.8 years in 146 patients with open globe injuries sustained at work, similar to this study.

The injuries in this study peaked in June. This was in accordance with a study evaluating open globe injuries from northwest Turkey, in which 33.7% of the injuries occurred in the workplace. They noted peaks in July and June.^17^ In southern Turkey, most of the penetrating eye injuries also took place in summer.^23^ Other studies have also reported that the majority of both work-related and non-work-related eye trauma occurred during summer months.^1,24^ In a large series of work-related eye injuries, the injuries peaked after lunch between 13:00 and 14:00.^21^ In contrast, another study reported that most work-related injuries occurred from 16:00 to 18:00.^1^ In the present study, the injuries peaked between 12:00 and 13:00. The second peak was between 14:00 and 15:00. A previous study reported that most of the work-related open globe injuries occurred at 10:00 to 11:00 and 15:00 to 16:00,^22^ which was corroborated by another study.^25^ It seems that the injuries usually occur around noon and afternoon. These variations may be related with working shifts or timing of lunch or the hazard of tasks performed in these time intervals. Various studies have documented metallic objects as the most common reported cause of injury in occupational eye injuries, with nails constituting the higher percentages.^1,22,26^ In the present study, miscellaneous metallic foreign bodies were the most common cause of injury, followed by iron particles and nails. These may be attributed to the industrial sectors in which the patients worked. Metalwork was the most common sector (38.2%) in this study. Most of the patients (86%) in the present study presented to emergency services within 12 hours after the injury, similar to another report of work-related open globe injury.^22^ The time between injury and presentation may be critical to achieve favorable outcome of primary repair.^27,28^

In this study, 78% of the patients did not wear protective eyewear. This is consistent with the findings of other studies.^20,29^ A study identifying risk factors for occupational eye injuries observed a 50% reduction in the incidence rate of eye injury among workers wearing protective eyewear compared to those who did not.^30^ Therefore, it may be useful to study what factors have an impact on use of protective eyewear both in Turkey and worldwide.

The clinical outcomes of the occupational traumas seen in the present study varied. Evisceration was performed in 4 eyes and enucleation in 1 eye. Similar rates of enucleation have been reported after work-related open globe injuries.^22,26^ In our study, two-thirds of the eyes that had injuries involving all 3 zones and one-third of the eyes that had injuries involving zones II and III resulted in phthisis bulbi, evisceration and enucleation. These findings suggest that large injuries and posterior segment involvement were related with poor visual outcomes.

In another study including 43 patients with occupational open globe injuries, the final VA was below 6/60 in 67% of the patients.^29^ In a study of nail-gun-induced open globe injuries, 86% were work-related, and 40.6% of the patients had a final VA below 20/200.^31^ Bauza et al.^26^ reported a final VA below 20/200 in 37.2% and Kanoff et al.^22^ reported that 25.9% of their patients had a VA below 20/200. In the present study, 33.3% of the patients had a final VA below 3/60. The relatively higher percentage of blindness in this study may be due to the fact that our department is the only tertiary center that receives complicated cases from a large industrialized region. Work-related open globe injuries may have severe consequences such as visual impairment and blindness among the young male worker population in industrialized areas. Nearly half of occupational open globe injuries result in visual impairment and blindness.

## CONCLUSION

Workers suffering work-related injuries may need several surgeries, and are unable to work during the treatment and rehabilitation period. Most of them lose their job or retire. Those who continue to work are usually monocular, which may increase their exposure to another occupational injury. These situations may cause financial, social and psychological burden on the workers, their families, employers and society. To avoid these burdens, simple protective measures such as use of protective eyewear or regular checking of working hours should be taken, especially in small-scale industrial sectors. Education of both workers and employers about protective measures and occupational injuries may raise their awareness.

### Ethics

Ethics Committee Approval: Retrospective study, Informed Consent: Retrospective study.

Peer-review: Externally peer-reviewed.

## Figures and Tables

**Table 1:**
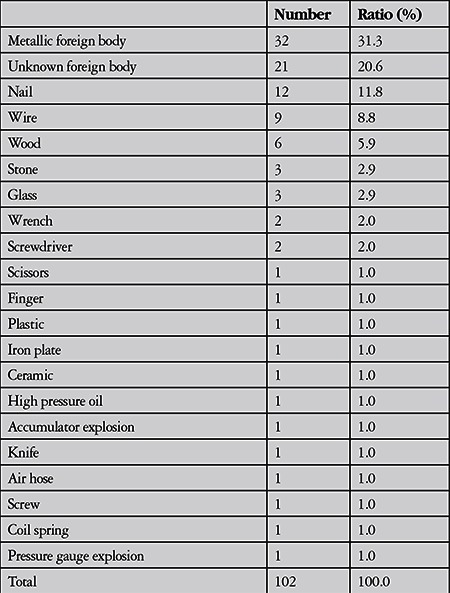
Causative objects of the work-related open globe injuries

**Table 2:**
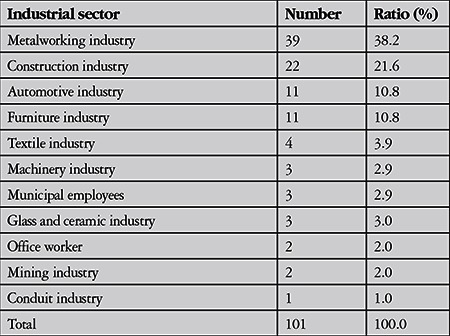
Industrial sectors of work-related open globe injuries occurred

**Table 3:**
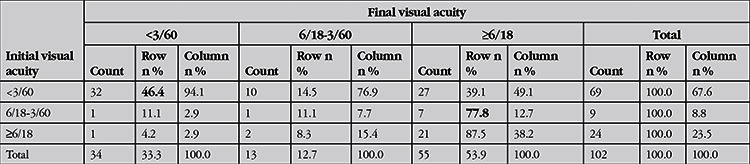
The initial and final visual acuities of the patients and categories of visual impairment according to World Health Organization criteria^13^

**Table 4:**
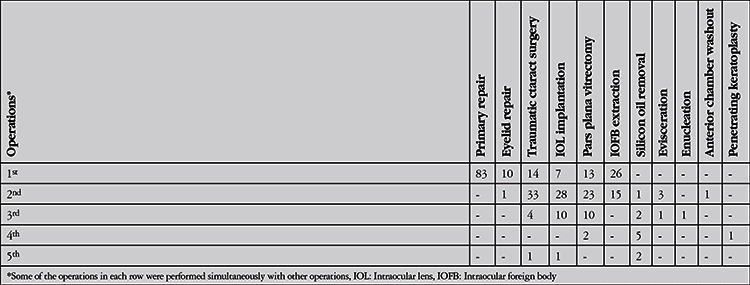
Primary and additional surgeries performed after primary repair

**Table 5:**
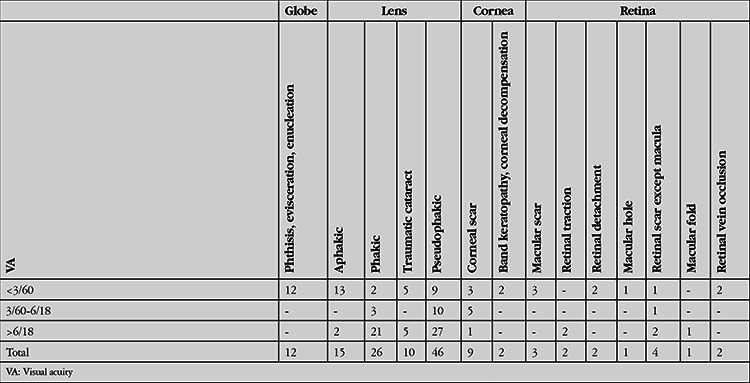
The final clinical outcomes of patients according to World Health Organization visual acuity categories
